# A Proposal for a Robust Validated Weighted General Data Protection Regulation-Based Scale to Assess the Quality of Privacy Policies of Mobile Health Applications: An eDelphi Study

**DOI:** 10.1055/a-2155-2021

**Published:** 2023-12-22

**Authors:** Jaime Benjumea, Jorge Ropero, Enrique Dorronzoro-Zubiete, Octavio Rivera-Romero, Alejandro Carrasco

**Affiliations:** 1 Department of Electronic Technology, Universidad de Sevilla, Sevilla, Spain

**Keywords:** participatory health informatics, mHealth apps, privacy policies, assessment scale, GDPR

## Abstract

**Background** Health care services are undergoing a digital transformation in which the Participatory Health Informatics field has a key role. Within this field, studies aimed to assess the quality of digital tools, including mHealth apps, are conducted. Privacy is one dimension of the quality of an mHealth app. Privacy consists of several components, including organizational, technical, and legal safeguards. Within legal safeguards, giving transparent information to the users on how their data are handled is crucial. This information is usually disclosed to users through the privacy policy document. Assessing the quality of a privacy policy is a complex task and several scales supporting this process have been proposed in the literature. However, these scales are heterogeneous and even not very objective. In our previous study, we proposed a checklist of items guiding the assessment of the quality of an mHealth app privacy policy, based on the General Data Protection Regulation.

**Objective** To refine the robustness of our General Data Protection Regulation-based privacy scale to assess the quality of an mHealth app privacy policy, to identify new items, and to assign weights for every item in the scale.

**Methods** A two-round modified eDelphi study was conducted involving a privacy expert panel.

**Results** After the Delphi process, all the items in the scale were considered “important” or “very important” (4 and 5 in a 5-point Likert scale, respectively) by most of the experts. One of the original items was suggested to be reworded, while eight tentative items were suggested. Only two of them were finally added after Round 2. Eleven of the 16 items in the scale were considered “very important” (weight of 1), while the other 5 were considered “important” (weight of 0.5).

**Conclusion** The Benjumea privacy scale is a new robust tool to assess the quality of an mHealth app privacy policy, providing a deeper and complementary analysis to other scales. Also, this robust scale provides a guideline for the development of high-quality privacy policies of mHealth apps.

## Introduction

Health services are undergoing a transformation in which patients take a more active role in their care, moving away from the traditional paternalistic model. This new role is the core of the participatory medicine, which identified this transformation as collaboration, empowerment, and shared decision-making about health. 1 Technology has a key position in this transformation and the term of participatory health informatics has emerged. 2 The use of technology to improve health care is now not restricted to health care institutions but used in a global way. The term “connected health” has been proposed by Caulfield and Donnelly as a health management model, in which patient data are shared in such a way that a patient can proactively receive medical care. 3 Among these technologies transforming health services, mobile technologies have been a subject of debate in the scientific literature in recent years, 4 being applied to multiple use cases. 5 The use of mobile technologies in the health domain is also called mobile Health (mHealth). Capabilities and characteristics of these mobile devices allow the implementation of several relevant health-related functionalities such as remote and real-time tracking of individual's conditions, adherence monitoring, real-time feedback, motivational messages, enhanced communications, etc. These functionalities enable the provision of personalized patient-centered health care services with a high cost-effectiveness. 6 7 8 9 Unfortunately, the quality of those mHealth solutions is not always high enough to reach this effectiveness and ensure a high adoption and adherence. 10 11 12 Assessment of digital health solution quality is a relevant topic in the Participatory Health Informatics field. Considering the technical specification ISO/TS 82304–2, “Health software—Health and wellness apps—Quality and reliability,” mHealth app quality is a multidimensional concept, 13 where privacy is included as one of the factors. The ISO/TS 82304–2 defines a set of factors that must be considered in the evaluation of the quality of an mHealth app. These factors are grouped into four domains: healthy and safe, easy to use, secure data, and robust build. The first domain includes items related to health requirements, health risks, ethics, health benefit, and societal benefit. The second one is composed of items related to accessibility and usability. The third domain consists of items related to privacy and security. Among other components, privacy statements and policy are assessed. In this study, we focus on privacy, particularly in the evaluation of the quality of a privacy policy. One of the data privacy strategies is to properly inform users regarding the personal information collected, saved and shared with others, and the personal data treatments. A privacy policy is considered the appropriate tool to provide this information to readers and potential users. The last domain included in this standard consists of items related to technical robustness and interoperability.

The privacy of the data handled by a mobile application is a recurring topic in the literature. Thus, for example, privacy concerns are one of the important issues for cancer patients who use mobile applications for self-management. 14 15 16 Likewise, Giunti et al 17 show how multiple sclerosis patients are especially concerned about the use of their personal data and who has access to them. In some of these studies, participants expressed their wishes to receive information regarding data privacy such as collected data and data sharing before the use of the mHealth app. This fact highlights the importance of providing appropriate privacy-related information. The appropriateness comprises not only the content itself but also the way the information is provided. Transparency and completeness of the information are required to build trustworthiness. Moreover, the information should be adapted to the potential users' literacy to enable adequate understanding and informed decisions. A high-quality privacy policy must consider these aspects to allow potential users to self-manage privacy when using the mHealth app and to prevent the increase of health disparities.

One of the strategies to improve privacy is to inform users about all relevant privacy issues regarding the use of the solution. Most of the privacy-related questions included in the ISO/TS 82304–2 technical specification are focused on providing information to the user. Thus, a privacy policy that shows all privacy-related information to the user is a key component of mHealth applications. The privacy policy in a health app presents information to the users on relevant aspects regarding the privacy of their data, such as the type of personal data collected by the app, the purpose of data processing, the establishing of a legal basis, and many other aspects. 18 In the field of mHealth, some authors have studied privacy policies in apps, since privacy policies represent the contract by which the data controller agrees to maintain the user privacy. Some papers have analyzed privacy policies in apps from different health conditions, like headache, 19 chronic insomnia, 20 depression, 21 blood pressure and diabetes, 22 cancer, 23 and mHealth apps in general. 24 25 26 The existence, 27 28 content, 19 20 21 23 24 25 26 29 30 31 32 33 34 and even the legibility of a privacy policy 22 26 34 35 were studied by researchers. Results about the existence of a privacy policy were varied. Some studies stated that 90% of the apps had a privacy policy, 25 27 others concluded that between 69 and 75% of the apps had a privacy policy. 23 32 36 Other authors stated than less than half the apps had a privacy policy. 21 26 Moreover, some authors tried to assess privacy policies according to different criteria. Some of them checked if the apps met the criteria they had previously defined, while others obtained a score for every app, creating scoring systems according to several items. 19 20 21 27 28 30 31 32 37 38 In this context, the existence of a legal regulatory framework that is capable of protecting the privacy of app user data is especially important. These legal frameworks grant users with new rights and force companies to deploy procedures to protect such rights. In the health domain, the United States pioneered, with the appearance of the Health Insurance Portability and Accountability Act (HIPAA) in 1996. The HIPAA regulations prohibited the disclose of medical information to anyone without the consent of the patient. 39 In 2009, the Health Information Technology for Economic and Clinical Health Act (HITECH Act) extended these responsibilities to the digital environment. 40 However, the FTC (Federal Trade Commission) noted that health apps, which can track everything from heart health to fertility or sleep data, increasingly collect sensitive and personal data from their users. Thus, FTC has warned health app developers to comply with Health Breach Notification Rule. These apps must ensure they secure the data they collect, which includes preventing unauthorized access to such information. 41 On the other hand, in Europe, in May 2018 a European Regulation, the General Data Protection Regulation (GDPR), came into effect. 42 The GDPR is a turning point regarding data protection in the European Union (EU), since it harmonizes the legislation throughout the EU. Nevertheless, the GDPR implementation across the EU needs harmonization. On the other hand, GDPR not only applies to those responsible or in charge of data treatment established in the EU, but also in the case that the responsible offers products or services to people within the EU, or if they monitor their behavior. This means that, in contrast to what happens in the United States, where laws are sectorial, GDPR applies to any company processing any kind of personal data, including health-related data.

Thus, a good starting point when evaluating privacy in mHealth applications is to analyze the level of compliance with the GDPR or HIPAA of the privacy policies published by the developers. Surprisingly, not many of the previously mentioned studies considered legal frameworks as a direct source in their assessment of privacy policies. GDPR was considered in the studies of Papageorgiou et al, 24 Hutton et al, 30 Leigh et al, 20 while HIPAA was contemplated by Zapata et al, 28 Bachiri et al, 29 and Mense et al 43 In a scoping review conducted by ourselves, 18 we concluded that although there are some proposals to assess privacy policies in mHealth applications, these are heterogeneous and even not very objective. Trying to fill this gap, we proposed a methodology for assessing privacy policies based on the GDPR. 23 This methodology, specifically designed for privacy policies in mHealth applications, consists of verifying, as a checklist, not only the mere existence or not of certain items in the assessed privacy policy, but also verifying that information is given in a transparent way, avoiding vague descriptions. It is important to emphasize that GDPR mandates this information must be easy to understand. Also, our methodology provides a simple scoring method for privacy policies. We identified 14 GDPR-based items, and a 0–1 score or a 0–0.5–1 score was assigned, depending on the item. The score was assigned beyond the strict compliance with the GDPR, applying the principles of lawfulness, fairness, and transparency. Thus, we designed a user guide with specific instructions to assess each item. A privacy policy scoring 100 points would be fully compliant with the GDPR. Only Hutton et al 30 considered a similar scheme, but they only considered 4 of the 14 GDPR-based items. Other studies which considered GDPR also contemplated between two and four of the GDPR-based items. 19 24 25 Thus, our previously designed scale (“original scale,” hereafter) constitutes a unique way of assessing privacy policies according to GDPR. In this article, we explore the robustness of the original scale, identify new items, and assign weights for every item in the scale. As a result, the Benjumea scale is a definite robust tool to measure the quality of privacy policy, a key privacy component, of an mHealth app.

## Objectives

Following a participatory method, an eDelphi study, this article has three main objectives. The first one is to explore the privacy experts' perceived robustness of the items of our previous objective GDPR-based privacy policy assessment scale (original scale) for mHealth applications. The second objective is to identify potentially relevant new items to be considered in the privacy policy assessment and refine the original scale. Finally, as it has been pointed out in several studies, 18 the third objective is weight assignment to every item in the scale according to the importance perceived by privacy experts. The level of consensus of a privacy expert panel on the relevance of the items included in the scale may be used as a measurement of robustness of this scale. Weights for each item can be defined based on the level of experts' consensus. For this reason, we have followed a participatory research method, an eDelphi technique.

The Delphi method was developed by Dalkey and Helmer, of the RAND Corporation, in the 1950s. 44 45 This method aims to achieve consensus among experts on a topic. An expert panel is involved following a structured process that is organized into rounds. In each round, each expert is provided with controlled feedback, excepting in the first one, and then inquired to provide their opinions on the studied topic using a predefined questionnaire. The feedback is based on his/her individual opinion and the experts' opinions in the previous round and avoids the pressure to conform to the majority opinion. Experts in the panel keep anonymous, avoiding domination of the consensus process by dominant individuals. 46 The process is repeated iteratively until the stopping criteria is reached. The eDelphi variant is a Delphi method in which questionnaires are delivered through the use of technology such as a web form. To the best of our knowledge, there is not Delphi reporting guidelines specifically designed for mHealth, but the CREDES guidelines could provide us with a good alternative. 47 Detailed information on the concrete method used in the Delphi study is recommended by CREDES to ensure its reproducibility and validity. A summary of CREDES reporting recommendations may be found in Supplementary Appendix A (available in the online version).

The definite scale proposed in this article might be a robust tool to measure the quality of privacy policy, a key privacy component, of an mHealth app. Assessing the privacy of mHealth apps requires analyzing several components, apart from the privacy policy, and therefore, this scale could be part of a more complete analysis. This scale could support studies within Participatory Health Informatics aimed to assess the quality of mHealth apps identifying elements to be improved.

## Methods

### Study Design

A nonexperimental study is proposed applying a modified Delphi methodology. 47 48 We decided to follow the modified method because we had identified the initial discussion points in our previous work 23 and, therefore, our study started collecting opinions on these predefined voting questions. The traditional Delphi method normally starts identifying the items to be evaluated in the later rounds using open-ended questions. 44 A two-round Delphi process is used to ask a panel of privacy experts about their opinion on the relevance of certain items proposed in the assessment of privacy policies in mHealth applications. This study culminates the work started in Benjumea et al, 23 with the assignment of weights for the items of a GDPR-based scale. It also allows the definition of new items, and the assessment of the robustness of every item in the scale.

The 14 items in the scale were proposed in Benjumea et al 23 to assess privacy policies, according to GDPR. This assessment should be understood as giving the users clear, easy to understand, information, and is implicit to GDPR. A brief description of the items in the scale is shown in Table 1 .

**Table 1:** Summary of GDPR-based scale items used in the assessment

Item identifier	Brief description
I1	Identity of data controller
I2	Identity of the representative
I3	Data protection officer details
I4	Purposes for the processing
I5	Legal basis for the processing
I6	Legitimate interests from controller
I7	Recipients (or categories) of the personal data
I8	Transfers to non-European Union countries
I9	Period for which data will be stored
I10	Existence of data subject's rights
I11	Existence of right to withdraw consent
I12	Right to lodge a complaint with a supervisory authority
I13	Obligation to provide personal data
I14	Existence of automated decision making or profiling

Abbreviation: GDPR, General Data Protection Regulation.

It was possible that new items were presented to the experts in Round 2 after their suggestions in Round 1.

In this study, the level of consensus for each item was reached when the interquartile range (IQR) was equal to or less than 1, since using IQR to check consensus is widely used in the literature. 49 If consensus was not found in some item, stability was checked using the Wilcoxon signed rank test of stability. Consensus and stability are only searched for compulsory items in the scale (i.e., those which were proposed in our previous work and are required by GDPR), and not for new items. We made a prespecification for two rounds as we considered that a third round could lead to cognitive fatigue of the experts. 50

### Selection Criteria and Recruitment

The panel of privacy policy experts was selected according to the following inclusion criteria: (1) being over 18 years, (2) agreeing to participate in the study, and (3) having experience in the application of privacy in the technological domain. Examples of participant profiles are: Data Protection Officers (DPOs), experts from the academic field, and app developers with some expertise on the GPDR.

For recruiting experts, a purposive sampling was used in this study. Specific data from possible participants were obtained from the research team professional network and from publicly accessible sources (mainly, but not limited to, from web pages). A first contact with the experts was made to explain the study to them. In case of being interested, some data from the experts were requested. First name, last name, email, position, and institution were eventually collected from the participants. These data were needed to build a well-balanced list of potential participants, but no personal information was used for the research. Participation in this study was voluntary, participants had no costs due to her/his participation, and no incentives for participation were offered. There were no risks, neither physical nor psychological, in the participation in this study.

The selected experts were invited to participate in the eDelphi study via email. Those who accepted were sent an email with a Participant Information Sheet (PIS) with all the aspects of the study. The email also included a link to Round 1. The participants were informed that, by completing the questionnaire, they were giving consent to participate in the study.

### Round 1

Round 1 took place between May 4, 2021 and May 24, 2021. A total of 22 experts received a link to Round 1 questionnaire, which was created using Microsoft Forms. The accessibility to the questionnaire in the Windows operating system was tested using Microsoft Edge, Firefox, and Google Chrome browsers, while accessibility in the MAC OS operating system was tested using the Safari browser. First and last name, email, position, and institution were collected. This information was gathered to obtain a well-balanced list of participants. Also, the information was needed for statistical proposes. No personal information was disclosed. Collecting email also prevents duplicated questionnaires. In case a participant filled out several questionnaires, the last one would be considered in the analysis.

In Round 1 questionnaire, the opinion of the participants was asked about the importance of the presence of 14 items in the privacy policy documents, precisely those that were present in our original scale.

The questions in the questionnaire were answered sequentially, and always in the same order. There was an adaptation in the questionnaire: it could not be opened until the participant gave his/her consent, but there was a link to view the questionnaire questions before giving consent. The questionnaire consisted of two screens or sections. Section 1 consisted of the information to the participant and the collection of his/her consent. Section 2 consisted of the questionnaire itself. The answers to all the questions were required, except for an open-ended question, where the experts could suggest new items. All the required fields were controlled by Microsoft Forms itself. The answers to the questionnaire were collected and processed using Microsoft Excel.

The user could see and modify his/her responses at any time before sending the questionnaire. Every item was evaluated according to its relevance using a 5-point Likert scale with extremes labeled (1, “Not important”; 5, “Very important”). Additionally, the experts were asked to point out more items that, in their opinion, should be used to assess privacy policies, so the questionnaire included a blank space at the end. A reminder was sent to the experts that had not filled in the questionnaire on May 13. Round 1 questionnaire and the changes introduced in it for Round 2 are shown in Supplementary Appendix B (available in the online version).

### Round 2

Round 2 took place between June 21, 2021 and July 16, 2021. All the experts that participated in Round 1 received a link to Round 2 questionnaire, which was also created using Microsoft Forms (Office 365), with the same features as Round 1 questionnaire. More items were added to Round 1 questionnaire to build Round 2 questionnaire, according to expert suggestions in Round 1. These items were proposed after using a simplified thematic analysis. 51 Researcher J.B. coded and analyzed expert suggestions, grouped them into common topics and categorized them, following the consensus with the rest of researchers.

During Round 2, a comparison of their responses in Round 1 and aggregated statistical data were generated and sent to participants. Customized PDF files containing Round 1 answers of each participant were generated in a semi-automated approach. An example of the PDF files can be found in Supplementary Appendix F (available in the online version). These files were sent by email to each participant, together with a link to a new questionnaire containing both the original 14 items and the new tentative items that could emerge from the open answers of Round 1 questionnaire.

In addition, they were asked to reassess the importance of these items, along with the new items suggested by experts in Round 1. A reminder was sent to the experts that had not filled in the questionnaire on July 6.

Tentative items would be eventually added to the scale if, at least, 80% of the experts rated the item as either 4 or 5. This criteria for inclusion has been widely used in the literature. 37 52 53 Compulsory items are considered robust with the same criteria, although they should not be eliminated from the scale as, otherwise, privacy policy would not comply with the GDPR. Finally, a weight is assigned to every item according to the obtained median of the scores in Round 2. If median equals to 5, the item is considered “very important,” and a weight of L1 = 1 is assigned to it. On the contrary, if median equals to 4, the item is considered “important,” and a weight of L2 = 0.5 is assigned to it.

### Ethical Considerations

*Ethical approval* : this study was reviewed and approved by the Andalusian Ethical Committee of Biomedical Research on March 30, 2021 with id. 0355-N-21. The committee is located at “CEI de los Hospitales Universitarios Virgen Macarena—Virgen del Rocío de Sevilla, Avda, Dr. Fedriani, 3 - Unidad de Investigación 2ª planta Sevilla 41071, Spain.” Also, the DPO of the Universidad de Sevilla supervised the processing of personal data involved in this study. 54

*Informed consent:* all participants received a PIS including all relevant information about this research study and how to contact with the research team. All participants agreed to participate in the project and gave their consent for data processing in accordance with our privacy policy. Participants were not able to continue their participation unless a checkbox was checked.

## Results

### Expert Panel

Initially, 27 experts were invited and accepted to participate in the study and 22 of them accessed and filled in Round 1 questionnaire. Round 2 questionnaire was sent to the 22 experts that filled in Round 1, and was completed by 19 experts. A summary is shown in Table 2 .

**Table 2:** Expert panel summary

Demographic criteria	Number of experts ( *n* = 19)
Gender	
Male	16 (84.21%)
Female	3 (15.79%)
Sector	
Public	11 (57.89%)
Private	8 (42.11%)
Position	
Lawyers working in privacy	7 (36.84%)
Data protection officers	5 (26.32%)
Academia	5 (26.32%)
IT security with knowledge in privacy	2 (10.52%)

Abbreviation: IT, information technology.

### Round 1

**Fig. 1:**
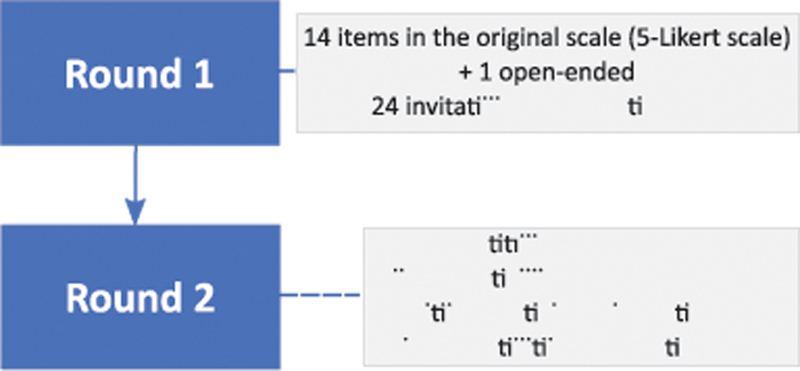
Delphi process.

The modified Delphi process is represented in Fig. 1 . All participants in Round 1 filled in the questionnaire with their evaluation of the initial 14 items. Results after Round 1 can be found in Supplementary Appendix C (available in the online version).

The open-ended question to suggest new items was filled in by 14 of 22 (63.6%) participants in Round 1. Some participants made similar suggestions using other wording. Additionally, some participants made more than one suggestion, and even some of the participants used this field to point out slight errors in the definition of the items. These suggestions were categorized as shown in Table 3 . The suggestions from the expert panel were considered as tentative items to be assessed in Round 2. These new items could be eventually added to our scale after Round 2, according to expert criteria. Tentative items were labeled from T1 to T9.

**Table 3:** Tentative items' categorization and definition

Category (item suggested)	Tentative item definition
Purposes for the processing	T1. Regarding the purposes for the processing (item I4), what characteristics of the purposes for the processing should be included?
Types of data	T2. Information about collected data (or categories of data).
Exercise rights within web	T3. Possibility to exercise user's rights within the web.
Data Processing Impact Assessment (DPIA)	T4. Access to the DPIA document, if available.
Security measures	T5. Information about deployed security measures.
Algorithms in automated decision making	T6. Disclosure of the algorithm used for automated decision making.
Certifications	T7. Information about certifications (ISO27001, ISO13485 o equivalent).
Last update	T8. Last update date of the privacy policy.
Normative	T9. Reference to data protection normative used to build the privacy policy.
Additional comments	Not applicable.

It is important to emphasize that all the suggestions were included for expert analysis in Round 2. Only suggestions that did not refer to a single item or piece of information that could be added to a privacy policy were discarded. Additional comments were used to refine the wording of some items.

Tentative items from T2 to T9 (see Table 3 ) were presented to experts in a 5-point Likert scale, where each item was evaluated according to its relevance (1, “Not important”; 5, “Very important”). Meanwhile, the intention with T1 was not creating a new item, but rephrasing item I4. I4 deals with the purpose for the data processing, but the level of details with which the purposes of the processing should be described was not included. Thus, T1 was the following multiple-choice question: “To what extent should the purposes for the processing be described?” The possible answers included the options “General description of the purposes for the processing,” “Specific description of the purposes for the processing,” and “Potential benefits to the user and to the data controller.” Thus, item I4 will be reworded according to the results obtained for item T1 in Round 2.

### Round 2

The files with the statistical data and answers of each participant were sent by email to each participant in Round 2 ( *n* = 19), together with a link to a new questionnaire containing both the original 14 items (I1 to I14) and the new tentative items (T1 to T9). Table 4 shows the aggregated results for all the items, evaluated with a 5-point Likert scale. More detailed data can be found in Supplementary Appendix D (available in the online version).

**Table 4:** Round 2 results for Likert-scale items

Item identifier	Median	% Ratings ≥4	IQR
I1	5	100.00%	0
I2	4	63.16%	2
I3	5	100.00%	0
I4	5	100.00%	0
I5	5	100.00%	0
I6	5	94.74%	1
I7	5	100.00%	0.5
I8	5	94.74%	1
I9	4	89.47%	1
I10	5	89.47%	1
I11	5	94.74%	1
I12	4	84.21%	1
I13	4	89.47%	1
I14	5	94.74%	1
T2	5	94.74%	1
T3	4	84.21%	0.5
T4	3	42.11%	2
T5	4	52.63%	1
T6	3	47.37%	1
T7	3	42.11%	1
T8	4	57.89%	2
T9	3	42.11%	2

Abbreviation: IQR, interquartile range.

According to the values of the IQR, consensus was achieved for all the items, except for I2, T4, T8, and T9. T4, T8, and T9 had a IQR of 2 but, also, the % Ratings ≥4 are, respectively 42, 58, and 42%. As our inclusion criteria is % Ratings ≥4 greater or equal 80%, we stopped further analysis because we considered they would not reach the 80% limit to be included. It seemed unnecessary to check consensus with items that would be discarded anyway. As for item I2, it was necessary to check its stability using the Wilcoxon signed rank test of stability. The calculated *p* -value for item I2 was *p* = 0.4689. As *p* >0.05, null hypothesis is accepted, so there was no difference between both Delphi rounds, regarding item I2. Therefore, all compulsory items reached consensus and/or stability in Round 2.

Item I4 must be reworded, as suggested by some of the experts, according to item T1 results. Since item T1 was assessed with a multiple-choice question, results are presented in a different way, as shown in Table 5 . Participants could select one or more answers for this item.

**Table 5:** Item T1 results

Answer	Number of times selected by participants ( *n* = 19)
General description of the purposes for the processing	9
Specific description of the purposes for the processing	17
Potential benefits to the user and to the data controller	9

Finally, regarding weight assignment, and based on the results presented in Table 4 , higher weights were assigned to those items that have been considered “very important” (median 5) at the expense of those considered “important” (median 4). Table 6 shows the weight assignment. L1 means a high weight, while L2 means a low weight. We propose values of L1 = 1 and L2 = 0.5.

**Table 6:** Weight assignment

Items with a weight of L1	I1, I3, I4, I5, I6, I7, I8, I10, I11, I14, T2
Items with a weight of L2	I2, I9, I12, I13, T3

## Discussion

A definite robust weighted GDPR-based scale to assess the quality of privacy policies in mHealth applications has been presented in this article, improving the original scale from our previous work. The Benjumea privacy scale will allow developers to build good privacy policies from the point of view of the GDPR and reviewers to assess the quality of these privacy policies. Thus, users will receive concise and clear information about privacy, which complies with the current regulations. Studies within the Participatory Health Informatics may use this scale as a measurement tool to conduct a deep analysis of the quality of mHealth apps. Based on a first approach to the development of the original scale, 23 we needed to evaluate its robustness. Thus, the aim of this article was twofold. First, to assess the robustness of a GDPR-based comprehensive scale. This objective was achieved by searching for consensus among a group of privacy experts. New items were added to the scale after the experts' suggestions. Second, weights were assigned to each of the items included in the definite scale, based on the experts' opinions. The Delphi process was considered the most appropriate method for gathering information from experts about the relevance of the selected items and their importance when evaluating the quality of mHealth app privacy policy regarding the GDPR. After two rounds, the modified Delphi process was stopped. A user guide, defining the use of new items and the items that have changed, is shown in Supplementary Appendix E (available in the online version). This user guide extends the guide of the original scale. Supplementary Appendix E (available in the online version) also shows how to calculate the final score for an assessed privacy policy, which is different from the scoring method in the original scale.

### Robustness

Quantifying the degree of consensus among the experts is an important component for performing a good Delphi data analysis and interpretation. In this study, we have used the IQR as a measure of the deviation of the opinion of an expert from the opinion of the whole panel. 49 52 A suitable IQR-based criterion to determine that there is a consensus among the experts is that IQR value is equal or less than 1 for a 5-point Likert scale. 55

Based on the IQR values for each item on the scale (see Table 4 ), the robustness of most of the items is supported by the consensus of the group of experts. This is a clear indication that the expert panel agrees with the current requirements of GDPR. However, item I2, which was included in the initial version of the scale, did not achieve a high level of consensus but the Wilcoxon signed rank test confirmed its stability. Item I2 is the only compulsory item with less of an 80% of ratings less than 4. As the item is a compulsory requirement of the GDPR, we propose not to exclude it from the scale, but to assign a low weight to it, as explained below.

As suggested by experts in Round 1, item I4 was reworded. Originally, this item dealt with the purposes of the processing. After checking experts' opinions, we conclude that a privacy policy must contain a specific description of the purposes of the processing, and not a general one. In the user guide of the original scale, it was not clear the level of details needed, so, following expert's opinions, we have updated the user guide of the original scale (see Supplementary Appendix E [available in the online version]) to make it clear that the purposes of the processing must be specific.

### New Items for the Scale

During the first round of the Delphi process, the experts identified new items that may be relevant when assessing the quality of the GDPR (see Table 3 ).

Among tentative items, there is a clear gap between finally selected items (T2 and T3) and the rest of the items. Most of the experts (more than 80%) assigned T2 and T3 a score of 4 or 5, which was our initial criteria to include them into the new scale. These items have been included in the user guide.

The inclusion of more tentative items could be argued, since they could provide more information to the users. From our point of view, not giving too much information is as essential as giving clear information in the privacy policy. Long privacy policies could have an undesirable effect on the users, refraining users from reading them. According to expert's opinion, discarded items are not important enough. It is important to emphasize that, for all discarded items, the proportion of experts that gave a score of 4 or 5 is less than proportion for the original item 2, which achieved 63.16%.

### Weight Assignment

In the first version of the scale, all the items contributed equally to the score to assess the quality of privacy policies. The original scale considered that every item had the same importance when evaluating them, regarding GDPR compliance. However, it is possible to consider that not all the measured items necessarily contribute with the same importance to the assessment of privacy policies. Thus, a weighted scale could be defined, assigning a weight to each item. The weight will be used in the successive computation of the score as each weight is multiplied for the corresponding individual value of the item.

Through the Delphi process, the expert panel has assigned a level of importance to each of the items on the scale. Therefore, it is possible to use this evaluation to assign weights to the items to reflect their impact of them on the score. Table 4 shows that all the original items have a median of 4 or 5. This fact is coherent, as these items are compulsory according to the GDPR, but also makes it harder to assign different weights, as all of them are important. We propose a weight L1 = 1 for “very important” items (median 5), and a weight L2 = 0.5 for “important” items. Then, items I1, I3, I4, I5, I6, I7, I8, I10, I11, I14, and T2 were assigned a weight of 1, while I2, I9, I12, I13, and T3 were assigned a weight of 0.5.

These results answer to the question if it is possible to assign different weights to items (I1 to I14) that are compulsory regarding the GDPR. It is perfectly possible to assume that if all items are compulsory, there should be no difference between the level of importance of each compulsory item. However, according to expert's opinion it is possible to assign two different levels of importance.

Supplementary Appendix E (available in the online version) shows how to calculate the score of a privacy policy with weighting applied.

### Comparison to Other Studies

Different studies have assessed privacy in mHealth apps. Even some of them have designed a scoring method for the assessment. 18 Surprisingly, only a few of them considered GDPR in their assessment. Hutton et al 30 considered GDPR and incorporated considerations from it into our framework. However, only some items from GDPR are represented in their developed scale. Papageorgiou et al 24 performed a GDPR compliance auditing procedure to determine whether the reviewed apps conform to the EU legal requirements. However, only four items are considered: user consent and its withdrawal, existence of a DPO, profiling, and transfer to third countries. Leigh et al 20 also used a few items from GDPR, and combined them with other sources, including a Delphi process with experts. Our study is the first in the matter that considers all the items that are contemplated in the GDPR, and then carries out a Delphi process to check with privacy experts if GDPR vision was correct and if important items were missing.

Only two studies have considered the possibility of using weights. 38 56 Brüggemann et al 56 define an information privacy risk index score, which allows users to either use the default weights or to set their own weights. However, how default weights were set is not explained. In Robustillo Cortés et al, 38 a stratified score was weighted according to an expert panel, after following a Delphi process. This method is in line with our way of defining the weights of the Benjumea scale. We consider that assigning weights to reflect the importance of each item in a privacy policy is an advance in their assessment.

### Limitations

This study has several limitations. First, although expert panel is intersectoral, including lawyers working in privacy, DPOs, people from academia, and IT security people with knowledge in privacy, it is not balanced regarding gender. Also, although GDPR is applicable in Europe, the panel involves only experts from Spain. An international expert panel might have assigned different relevance scores and proposed new items.

We designed a Delphi study involving only two rounds. Therefore, the stability of the scores assigned by participants to the tentative items could not be analyzed. Our decision was made to reduce the potential respondent fatigue and to avoid an increased dropout rate that may lead to a small sample size of participants responding to the questionnaire. However, a third round could provide us with the opportunity to assess the stability of the scores assigned by participants to the tentative items. Also, new items could have been reported by participants in the third round. However, these new items would have required additional rounds to assess experts' opinion consensus and stability. This fact may impact on the respondent fatigue as experts would have been asked to fill out longer questionnaires in the additional rounds potentially leading to an increased dropout rate.

Criterion used to include tentative items was 80% of the experts rated the item in round 2 as either 4 or 5. This criterion is widely used in the literature. However, we excluded two tentative items (T5 and T8) with more than 50% and less than 80% of the experts rating them either 4 or 5. Perhaps, further analysis, including a third round for these items, could have led to the inclusion of them. Anyway, as shown in Table 4 , T5 and T8 had a less “% Ratings ≥4” than I2 (the compulsory item with worse results) and only the median value is the same.

As tentative items were included only in one round, their stability was not evaluated. Therefore, the relevance scores of these tentative items could change in further rounds.

## Conclusion

In this article, we studied the robustness of a GDPR-based scale for the assessment of privacy policies in mHealth applications. We also studied weight assignment for the items of the scale. With these aims, we conducted a two-round modified Delphi process, where an expert panel assigned an importance to every item of the scale, using a 5-point Likert scale. Experts also suggested new items, which were evaluated in Round 2, for their possible inclusion in the privacy scale. After the Delphi process, the results showed a great robustness of the scale, and two new items were finally added to it. Moreover, weights were assigned to every item in the scale. The result is a definite robust, weighted, GDPR-based privacy scale, which has been named as Benjumea privacy scale. This scale provides a measurement tool to be used in studies focused on assessing the quality of mHealth apps within the Participatory Health Informatics field.
