# Supplementary material for: A Proposal for a Robust Validated Weighted General Data Protection Regulation-Based Scale to Assess the Quality of Privacy Policies of Mobile Health Applications: An eDelphi Study
Source: Methods Inf Med. 2023 Dec 22;62(05-06):154–64. doi: 10.1055/a-2155-2021 (PMC10878744; doi:10.1055/a-2155-2021)
Supplement: Supplementary file 1 — Supplementary Material [file 10-1055-a-2155-2021-s22020017.pdf]

Supplementary Appendix

Supplementary Appendix A Summary of CREDES reporting recommendations

Recommendation	Item #	Explanation	Reported on
Purpose and rationale	8	The purpose of the study should be clearly defined and demonstrate the appropriateness of the use of the Delphi technique as a method to achieve the research aim.<br>A rationale for the choice of the Delphi technique as the most suitable method needs to be provided	The purpose is reported in Abstract and the section Introduction, pages 1 and 2. Appropriateness and rationale could be found in the section Study Design, pages 3 and 4.
Expert panel	9	Criteria for the selection of experts and transparent information on recruitment of the expert panel, socio-demographic details including information on expertise regarding the topic in question, (non)response and response rates over the ongoing iterations should be reported	Panel expert information is reported in the section Selection Criteria and Recruitment, page 4.<br>Socio-demographic details and response rates are reported in the section Expert Panel, page 5.
Description of methods	10	The methods employed need to be comprehensible; this includes information on preparatory steps, piloting of material and survey instruments, design of the survey instrument(s), the number and design of survey rounds, methods of data analysis, processing and synthesis of experts' responses to inform the subsequent survey round, and methodological decisions taken by the research team throughout the process	Information about methods is reported on the section Round 1, pages 4–5, and the section Round 2, page 5.
Procedure	11	Flow chart to illustrate the stages of the Delphi process, including a preparatory phase, the actual "Delphi rounds," interim steps of data processing and analysis, and concluding steps	Flow chart is reported on the section Round 1, page 6.
Definition and attainment of consensus	12	It needs to be comprehensible to the reader how consensus was achieved throughout the process, including strategies to deal with nonconsensus	Consensus definition is reported in the section Study Design, page 4.<br>Strategies to deal with dissent and consensus are reported in the section Study Design, page 4.
Results	13	Reporting of results for each round separately is highly advisable to make the evolving of consensus over the rounds transparent. This includes figures showing the average group response, changes between rounds, as well as any modifications of the survey instrument such as deletion, addition, or modification of survey items based on previous rounds	Results of Round 2 are reported in the section Results, page 7.<br>Results of Round 1 are reported in ►**Supplementary Appendix C**.<br>Figures are reported in ►**Supplementary Appendix F**.<br>Modifications are reported in ►**Supplementary Appendix D**.
Discussion of limitations	14	Reporting should include a critical reflection of potential limitations and their impact of the resulting guidance	Limitations are reported in the section Limitations, pages 8–9.
Adequacy of conclusions	15	The conclusions should adequately reflect the outcomes of the Delphi study with a view to the scope and applicability of the resulting practice guidance	Conclusions are reported in the section Conclusion, page 9.
Publication and dissemination[a]	16	The resulting robustness and redefinition of scale should be clearly identifiable from the publication.	Robustness and redefinitions are reported in the section Discussion, pages 7–8

[a]We have adapted this item to the study objectives.

 © 2023. The Author(s).

Supplementary Appendix B

Round 1 Questionnaire (Translated from Spanish)

Categorization of the relevance of components of privacy policies.

Thank you very much for participating in this study, this page shows basic information about the project. However, you can access the Participant Information Sheet at https://uses0-my.sharepoint.com/:b:/g/personal/jaimebm_us_es/EbIFkEg28eZAvXQJFyLaspsB6eeNeKifGSGPZQHIubIH7A?e=qwEt3T.

If you want to see the content of the survey before continuing, you can see it at https://uses0-my.sharepoint.com/:b:/g/personal/jaimebm_us_es/EdJL1oxgG65Lup3whfXif_8BMRiVWLfyfy4h-FLCXW6V9w?e=TClpYm.

Your participation in this study consists of two phases:

- In the first one (this questionnaire) you must fill in a questionnaire, in which your opinion will be asked about the importance of the presence of certain items in the privacy policy documents in mobile health applications. These items are indicated in the Article 13 of the General Data Protection Regulation (GDPR). Additionally, you will be asked to point out, if you wish, any other item that, in your opinion, should be used to assess privacy policies.
- In the second round (an email containing a link will be sent to you in the coming weeks), you will be shown aggregated statistical data from the answers of other participants in the study, together with a comparison with your previous round answers. You will be asked again to rate the importance of these items together with others that could be identified in the previous round.

Remember that your participation in this study is voluntary and, by sending this form, you give your consent for your personal data to be processed, in accordance with the information clause, available at https://sic.us.es/sites/default/files/pd/cievaluacionpolprivacidad.pdf.

If you need more information, you may contact Alejandro Carrasco Muñoz (acarrasco@us.es)

Contact and demographic data
Enter your personal data below (all fields are required)
Surname:
Name:
Position:
Institution:
Email address (it will be used throughout the study):

By checking the following box, you agree to participate in the project and give your consent for your data to be processed in accordance with our privacy policy: [ ]

Assessing the Importance of Certain Items in Privacy Policies

Point out the relative importance, in your opinion, of the presence of certain information (items) in the privacy policies of mobile health applications. When answering this questionnaire, keep in mind that, beyond strict compliance with the GDPR (and, specifically, article 13), you must give your opinion on the importance of these items.

Value the importance that the following information appears in the privacy policies of mobile health applications:

Item identifier	Brief description	1 Not important	2 Slightly important	3 Moderately important	4 Important	5 Very important
I1	Identity of data controller					
I2	If applicable, identity of the representative					
I3	Data protection officer details					
I4	Purposes for the processing					
I5	Legal basis for the processing					
I6	If applicable, legitimate interests from controller					
I7	If applicable, recipients (or categories) of the personal data					
I8	If applicable, information about transfers to non–European Union countries					

(*Continued*)

 © 2023. The Author(s).

(*Continued*)

Item identifier	Brief description	1 Not important	2 Slightly important	3 Moderately important	4 Important	5 Very important
I9	Period for which data will be stored					
I10	Information about the existence of data subject's rights					
I11	If applicable, information about the existence of right to withdraw consent					
I12	Information about the right to lodge a complaint with a supervisory authority					
I13	If applicable, information about the obligation to provide personal data					
I14	Information about the existence of automated decision making, including profiling					

Is there any other item that you think should appear in the privacy policy documents in mobile health applications? If so, use the space below to describe it, as well as a brief detail of the reasons why you are making your proposal.

Round 2 Questionnaire (Translated from Spanish)
Categorization of the relevance of components of privacy policies (Round 2).

Thank you very much for participating in the second round of this study. Remember you can access the Participant Information Sheet at https://uses0-my.sharepoint.com/:b:/g/personal/jaimebm_us_es/EbIFkEg28eZAvXQJFyLaspsB6eeNe-KifGSGPZQHIubIH7A?e=qwEt3T.

Your participation in this study consists of two phases:

- In the first one (already completed) you filled in a questionnaire, in which we asked your opinion about the importance of the presence of certain items in the privacy policy documents in mobile health applications. These items are indicated in the Article 13 of the General Data Protection Regulation (GDPR). Additionally, you were asked to point out, if you wished, any other item that, in your opinion, should be used to assess privacy policies.
- In the second round (this one), we have sent you an email with aggregated statistical data from the answers of other participants in the study, together with a comparison with your previous round answers. You are now asked to rate again the importance of these items together with others that have been identified in the previous round.

Remember that your participation in this study is voluntary and, by sending this form, you give your consent for your personal data to be processed, in accordance with the information clause, available at https://sic.us.es/sites/default/files/pd/cievaluacionpolprivacidad.pdf.

If you need more information, you may contact Alejandro Carrasco Muñoz (acarrasco@us.es).

Email address (use the same email you used in round 1):

Value the importance that the following information appears in the privacy policies of mobile health applications:

Item identifier	Brief description	1 Not important	2 Slightly important	3 Moderately important	4 Important	5 Very important
I1	Identity of data controller (including name, postal and electronic address)					
I2	If applicable, identity of the representative (including name, postal and electronic address)					
I3	Data protection officer details					
I4	Purposes for the processing					
I5	Legal basis for the processing					
I6	If applicable, legitimate interests from controller					

 © 2023. The Author(s).

(*Continued*)

Item identifier	Brief description	1 Not important	2 Slightly important	3 Moderately important	4 Important	5 Very important
I7	If applicable, recipients (or categories) of the personal data (expected cessions of data)					
I8	If applicable, information about transfers to non-EEA countries (EU and Liechtenstein, Iceland and Norway) or international organizations					
I9	Period for which data will be stored					
I10	Information about the existence of data subject's rights (access, rectification, erasure, restriction of processing, objection to processing and portability) and how to exercise them.					
I11	If applicable, information about the existence of right to withdraw consent					
I12	Information about the right to lodge a complaint with a supervisory authority					
I13	If applicable, information about the obligation for the data subject to provide personal data and the consequences of not providing them.					
I14	Information about the existence of automated decision making, including profiling					

Regarding the purposes for the processing (item I4), what characteristics of the purposes for the processing should be included? (One or more options may be selected)

[ ] General description of the purposes for the processing.
[ ] Specific description of the purposes for the processing.
[ ] Potential benefits to the user and to the data controller.

Item identifier	Brief description	1 Not important	2 Slightly important	3 Moderately important	4 Important	5 Very important
T2	Information about collected data (or categories of data).					
T3	Possibility to exercise user's rights within the web.					
T4	Access to the DPIA document, if available.					
T5	Information about deployed security measures.					
T6	Disclosure of the algorithm used for automated decision making.					
T7	Information about certifications (ISO27001, ISO13485 o equivalent).					
T8	Last update date of the privacy policy.					
T9	Reference to data protection normative used to build the privacy policy					

Supplementary Appendix C Results after Round 1

Item identifier	Median	% Ratings $\geq$ 4	IQR
I1	5	100.00%	0
I2	4	63.63%	2
I3	5	100.00%	0
I4	5	100.00%	0
I5	5	95.45%	1
I6	5	95.45%	1
I7	5	100.00%	0.75
I8	5	90.90%	1
I9	5	86.36%	1
I10	5	90.90%	1
I11	5	100.00%	1
I12	4	77.27%	1
I13	4	86.36%	1
I14	5	95.45%	1

Abbreviation: IQR, interquartile range.

Supplementary Appendix D Round 2 questionnaire results

Item name	Not important	Slightly important	Moderately important	Important	Very Important
I1. Identity of data controller.	0	0	0	1	18
I2. Identity of the representative.	0	2	5	6	6
I3. Data Protection Officer (DPO) details.	0	0	0	2	17
I4. Purposes for the processing.	0	0	0	0	19
I5. Legal basis for the processing.	0	0	0	3	16
I6. Legitimate interests from controller.	0	0	1	6	12
I7. Recipients (or category of recipients) of the personal data.	0	0	0	5	14
I8. Transfers to non-EEA countries.	0	0	1	5	13
I9. Period for which data will be stored.	0	0	2	8	9
I10. Existence of data subject's rights.	0	0	2	5	12
I11. Existence of the right to withdraw consent.	0	0	1	6	12
I12. Right to lodge a complaint with a supervisory authority.	0	1	2	10	6
I13. Obligation to provide personal data.	0	1	1	8	9
I14. Existence of automatic decision-making or profiling.	0	0	1	5	13
T2. Information about collected data (or categories of data).	0	1	0	7	11
T3. Possibility to exercise user's rights within the web.	0	1	2	11	5
T4. Access to Data Privacy Impact Assessment document, if available.	1	5	5	7	1
T5. Information about deployed security measures.	0	3	6	8	2
	2	1	7	5	4

 © 2023. The Author(s).

(*Continued*)

Item name	Not important	Slightly important	Moderately important	Important	Very Important
T6. Disclosure of the algorithm used for automated decision making.					
T7. Information about certifications (ISO27001, ISO13485 o equivalent).	0	4	7	6	2
T8. Last update date of the privacy policy.	0	2	6	5	6
T9. Reference to data protection normative used to build the data privacy policy.	2	5	4	5	3

Supplementary Appendix E

New and changed items:

The following items have been reworded (item 4) and added (items 15 and 16) to the original user's guide[1] (►**Supplementary Tables S1**–**S3** [online only].

Supplementary Table S1 Item 4: Purposes for the processing

**Item number:** 4		
**Name:** Purposes for the processing		
**Short description:** The purposes for the processing must be specific.		
**Source:** GDPR mandates that the purposes for the processing must be explicit, somehow, in the privacy policy.		
**Score:** Sometimes this information is given but information is too generic, and this is a bad practice since data controller must provide specific information. For example, "We collect this information for the purpose of providing our service" does not give any detail about why the data controller needs the personal data. In this case, the score for this item is 0.5 points. If purposes are provided specifically, 1 point is given. If some purposes are explicit in the privacy policy but, at the same time, some wildcard such as "any other purpose..." score will be 0.5 points. If purposes are not mentioned, 0 points. Please notice that this information is, sometimes, scattered through the privacy policy. There is no penalty in this case.		
**Examples**	**1 point**	*Broadly speaking, we use personal information for purposes of administering our business activities, providing customer service and making available other products and services to our customers and prospective customers. Occasionally, we may also use the information we collect to notify you about new services and special offers we think you will find valuable.*
	**0.5 points**	*While using our Service, we may ask that you provide us with certain information that can be used to contact or identify you. Personally identifiable information may include, but is not limited to, your email address, full name, or other information ("Personal Information"). We collect this information for the purpose of providing our Service.*
	**0 points**	

 © 2023. The Author(s).

Supplementary Table S2 Item 15: Information about data (or categories of data) collected

<table>
<tr><td colspan="3">Item number: 15</td></tr>
<tr><td colspan="3">Name: Information about data (or categories of data) collected.</td></tr>
<tr><td colspan="3">Short description: The privacy policy must have explicit information about the data (or its categories) collected by the app.</td></tr>
<tr><td colspan="3">Source: This item is under consideration by a board of experts in privacy.</td></tr>
<tr><td colspan="3">Score: 1 point if this information is given, 0 points otherwise. Please note that it is not necessary to have this information in a separate paragraph or section.</td></tr>
<tr><td rowspan="2">Examples</td><td>1 point</td><td>WHAT WE COLLECT
We collect both personal and nonpersonal information from and about individuals who use the Services. “Personal information” is any information relating to an identified or identifiable natural person. “Nonpersonal information” may include technical information that does not identify an individual personally. We note that over time, nonpersonal information could become personal information through regulatory developments, technological advancements, or co-mingling with personal information. In particular, we collect the following information from and about you:
Information You Give Us. We may collect, store, and use personal information that you may voluntarily submit to us, including your name, postal address, email address, phone number, username, password, demographic information (such as your gender and occupation), date of birth, profile image, and contact preferences. We may also collect, store, and use certain health information that you elect to provide to us through the Services, including diagnosis information, symptoms, treatment information, and activity. In using the Services, you are free to skip any nonrequired questions or data fields that make you feel uncomfortable. You are also free to stop using the Services at any time.
Information Automatically Collected. We may collect certain information automatically, including, but not limited to, the type of mobile device you use, your mobile device’s unique device ID, the IP address of your mobile device, your mobile operating system, the type of mobile Internet browsers you use, and information about the way you use the Services, including the sections and features of the Services you viewed or used, and how long you spent on a particular section or feature.
Information We Get from Others. We may obtain information about you from other sources such as in connection with a medical study that you are participating in with a health care provider that encourages your use of our Services in connection therewith. We may add this to information we obtain from the Services.[2]</td></tr>
<tr><td>0 points</td><td></td></tr>
</table>

Supplementary Table S3 Item 16: Possibility to exercise user’s rights within the web

<table>
<tr><td colspan="3">Item number: 16</td></tr>
<tr><td colspan="3">Name: Possibility to exercise user’s rights within the web.</td></tr>
<tr><td colspan="3">Short description: An option to exercise user’s rights (for example, those mentioned in items 10, 11, and 12) directly within the web must exist.</td></tr>
<tr><td colspan="3">Source: This item is under consideration by a board of experts in privacy.</td></tr>
<tr><td colspan="3">Score: One point if the privacy policy offers this feature, zero points otherwise. Keep in mind that giving an electronic address is not enough for this item to be scored with one point. There must exist some kind of web form explicitly designed for this purpose.</td></tr>
<tr><td rowspan="2">Examples</td><td>1 point</td><td>A web form to exercise user’s rights exists.</td></tr>
<tr><td>0 points</td><td></td></tr>
</table>

Calculation formula for assessment:

Final score for a privacy policy is measured as a percentage of the total possible number of points available. This means that if an item gets a Not Applicable (N/A) score, this item must be ignored.

The calculation formula for our scale is as follows:

Gross Score = I1 + I3 + I4 + I5 + I6 + I7 + I8 + I10 + I11 + I14 + I15 + (I2 + I9 + I12 + I13 + I16) * 0.5

Final Score = Gross Score * (100/(13.5 – Weighted_value_of_items_with_NA)).

References

1 Benjumea J, Ropero J, Rivera-Romero O, Dorronzoro-Zubiete E, Carrasco A. Assessment of the fairness of privacy policies of mobile health apps: scale development and evaluation in cancer apps. JMIR Mhealth Uhealth 2020;8(07):e17134

2 chemoWave: for cancer patients. Accessed June 2022 at: http://chemowave.com/privacy/

 © 2023. The Author(s).

Supplementary Appendix F Table showing results of round 1 to participant (translated from Spanish)

Data for: XXXXXXXX

Below, you will find the results after first round of this study.

For each item, you will find, in the first column, the value you assigned to this item. The second column shows the average value for all participants.

Item name	Your value	Average value
1. Identity of data controller	5	4.91
2. If applicable, identity of the representative	4	4.00
3. Data protection officer details	5	4.82
4. Purposes for the processing	5	4.95
5. Legal basis for the processing	5	4.64
6. If applicable, legitimate interests from controller	4	4.55
7. If applicable, recipients (or categories) of the personal data	5	4.73
8. If applicable, information about transfers to non-European Union countries	4	4.55
9. Period for which data will be stored	5	4.36
10. Information about the existence of data subject's rights	5	4.55
11. If applicable, information about the existence of right to withdraw consent	5	4.64
12. Information about the right to lodge a complaint with a supervisory authority	5	4.09
13. If applicable, information about the obligation to provide personal data	5	4.32
14. Information about the existence of automated decision making, including profiling	4	4.64

 © 2023. The Author(s).
